# Technical Report: Efficacy and Safety of Low-Intensity Transcranial Magnetic Stimulation in the Remission of Depressive Symptoms in Patients With Treatment-Resistant Depression in Mexico

**DOI:** 10.7759/cureus.59612

**Published:** 2024-05-03

**Authors:** Cristina Rodríguez Hernández, Omar Medrano Espinosa, Raúl Sampieri-Cabrera, Alan R Oviedo Lara

**Affiliations:** 1 Psychiatry, Rome Psiquiatría Integral, Mexico City, MEX; 2 Department of Physiology, Faculty of Medicine, Universidad Nacional Autónoma de México, Mexico City, MEX; 3 Research, Nibbot International, Mexico City, MEX

**Keywords:** low intensity tms, major depression disorder, noninvasive neuromodulation, transcranial magnetic stimulation (tms), treatment-resistant depression (trd)

## Abstract

Transcranial magnetic stimulation (TMS) is a non-invasive neuromodulation technique that induces action potentials in the stimulated cortical area and has been approved by the Food and Drug Administration (FDA) for the treatment of major depressive disorder (MDD). The prevalence of MDD in Mexico almost tripled after the COVID-19 pandemic. In this study, we evaluated the safety and therapeutic effects of low-intensity TMS (Li-TMS) - characterized by inducing electric currents below the action potential threshold on the cerebral cortex - in 41 subjects diagnosed with treatment-resistant depression (TRD). A Li-TMS device dispensed repetitive magnetic pulses at 30 mT for 60 minutes during 20 sessions (once daily from Monday to Saturday) with the theta burst pattern. Our results suggest that Li-TMS is a safe therapy with antidepressant effects, demonstrated by the decrease in Beck Depression Inventory (BDI) scores and lessening of depressive symptoms.

## Introduction

Treatment-resistant depression, prevalence, and treatment in Mexico

Most of the definitions of depression emphasize that it is a medical condition characterized by a low mood, anhedonia, and deep feelings of restlessness and hopelessness persisting for more than two weeks and are related to a change in previous functioning. This mental disorder invariably entails mood alterations, such as sadness, irritability, emptiness, and loss of interest. Therefore, an accurate assessment of the degree of depression is essential to guide appropriate clinical interventions. Depression is classified into mild, moderate, or severe, depending on the number of symptoms, intensity, and impact on a person's function. This classification is crucial because the severity of depression determines the required treatment intensity [1]. In mild cases, a psychotherapeutic approach without medication may be appropriate, while in moderate and severe depression, antidepressants are usually prescribed in doses that correspond to the severity of symptoms. This strategy ensures personalized and effective care, minimizing the risks of overmedication or insufficient treatment [2]. These behavioral symptoms are associated with biological and neurovegetative features (including changes in sleep patterns, weight, energy levels, sexual appetite, and psychomotor activity), alongside cognitive features, such as self-distortion and altered perception of the world and future, accompanied by feelings of guilt and indecision [3-5]. The biological bases of this disease are complex and include neuronal and neuroglia alterations in specific areas of the brain. At the molecular level, this implies various biological processes that are not limited to the change in the homeostasis of monoamine neurotransmitters; instead, they also include a reduction in neurotrophic aid, metabolic dysfunction, altered immune response due to an increase in inflammatory response, oxidative stress, and mitochondrial dysfunction [6]. Ultimately, depression affects cerebral plasticity and synaptic function; these processes are involved in long-term functional damage, which may cause cognitive impairment [7]. Depression and neurodegenerative diseases, such as dementia, share key features, including dysfunctions in neurotransmission, accumulation of β-amyloid protein, alterations in the hypothalamic-pituitary-adrenal axis, neuroinflammation, and oxidative stress. These similarities suggest interconnected pathological mechanisms affecting both depression and neurodegeneration [8]. 

This mental disorder affects around 5% of the world's population and is considered one of the most common causes of the global burden of disease [9]. According to the World Health Organization (WHO), reports of depression and anxiety globally increased by approximately 25% after the COVID-19 pandemic. It is projected that depression will become the leading cause of the global burden of disease by 2030 [10-11]. In Mexico, according to a national survey conducted in 2022 called the Second Operative Diagnostic of Mental Health and Addictions, depression is the most prevalent mental illness, affecting 5.5% of the population [12]. Depression affects people of all ages, but women have approximately twice the risk of developing depression compared to men. This is due to sexual differences in the physiology of the brain that may make women more susceptible to major depressive disorder (MDD). These differences are linked to variations in the excitability of specific brain circuits, such as the circuit extending from the ventral hippocampus to the nucleus accumbens, which influence how depression-related behaviors manifest. Studies on these sexual differences are crucial for developing diagnostics and treatments that are sex-specific for MDD and other mood disorders [13]. Furthermore, the Health at a Glance report from the Organization for Economic Cooperation and Development (OECD) estimates that depression prevalence in Mexico has increased by around 71% compared to 2019, primarily due to the COVID-19 pandemic and economic crisis [14]. The economic impact of out-of-pocket spending on depression is substantial, affecting both individual and macroeconomic economies. High costs resulting from gaps in the detection and treatment of MDD reduce consumption and labor productivity, exacerbating the economic crisis. Investing in improved detection and treatment can be costly initially, but it translates into a cost-effective use of resources in the long term, improving quality of life and reducing the overall economic burden [15].

The therapeutic goal for treating depressive disorder is complete symptom remission, where the patient fully recovers psychosocial functioning with minimal or, if possible, no residual symptoms [16]. Despite the availability of multiple antidepressant drugs, only 60-70% of patients who tolerate pharmacological treatment adequately respond to monotherapy in first-line treatment [17-18]. The term ”treatment-resistant depression” (TRD) describes a medical condition in patients with MDD who do not respond adequately to two schemes of antidepressant drugs at an adequate dose within an expected period, typically around four weeks [19]. Pharmacological therapy for depression often fails due to genetic variations that affect patient response [20], highlighting the need for alternative therapies, such as transcranial magnetic stimulation (TMS). TMS offers direct modulation of cortical activity and has been proven effective in cases of TRD, also presenting a favorable safety profile. These features make TMS a valuable therapeutic option when conventional antidepressants are insufficient [21].

In addition to the current challenges in treating MDD, such as drug treatment resistance, dosing issues, inadequate diagnosis, and biological features, socioeconomic factors also play a significant role in accessing adequate treatment in developing countries. In Mexico, only 20% of the population with this mental illness seeks professional assistance, and of that number, only 50% have access to minimally adequate treatment, including drug medications or psychological/psychiatric therapies [22]. In addition, high stress levels are associated with an increase in depression symptoms, while social support is related to a reduction in the risk of depression. The adequate provision of treatment for depression should consider interventions that increase social support and address specific stress factors, especially in populations with social vulnerability. For example, during the pandemic, women in Mexico with high levels of stress experienced a significant increase in depressive symptoms (β: 2.13; p < 0.001) and a higher risk of depression (odds ratio (OR): 3.75; p < 0.001). For them, social support was associated with a reduction in the risk of depression (OR: 0.56; p = 0.037). These current issues and prevalence projections pose a significant challenge to mental health specialists in Mexico, as this condition already has a notable social impact [23].

TMS

TMS has been widely reported as a safe, non-invasive, and effective therapy when applied to treat different disorders of the central nervous system (CNS), particularly in cases where patients have shown little or no response to psychotherapies and/or pharmacological therapies (such as TRD) [24]. TMS involves delivering frequency and intensity-controlled magnetic pulses over a specific area on the subject's scalp. These repetitive magnetic pulses penetrate the cerebral cortex, inducing electric currents. When the intensity of the magnetic pulses is around 1-1.5 Tesla (T), they can generate an electric current sufficient to trigger an action potential (evoked potential). TMS has primarily been used to treat depression, especially in patients resistant to drugs (TRD), demonstrating significant therapeutic benefits leading to remission [21]. In addition, TMS has been explored for its therapeutic effects in various pathologies, including obsessive-compulsive disorder (OCD) [25], autism spectrum disorders (ASD) [26-27], attention-deficit hyperactivity disorder (ADHD) [28], Alzheimer's disease [29], anxiety [30], and tinnitus [31-32], among others, with robust scientific support.

Low-intensity transcranial magnetic stimulation (Li-TMS)

Li-TMS is a safe, non-invasive, and outpatient neuromodulation tool, which in contrast to high-intensity transcranial magnetic stimulation (Hi-TMS), uses magnetic fields in the order of milliTeslas (mT), that does not induce an action potential at the stimulated cortical area. For this reason, this technique was also known as subthreshold magnetic stimulation, and before the publication by J. Moretti and J. Rogers in 2022 [33], it was also known as pulsed electromagnetic field therapy (PEMF). Nonetheless, by reducing the magnetic field, it is possible to implement higher frequencies in these systems compared to those included in Hi-TMS devices (ranging from 1 to 600 Hz). Similar to Hi-TMS, Li-TMS can activate or deactivate neuronal activity depending on the frequency of the dispensed magnetic pulses (1-5 Hz is inhibitory, and above 5 Hz promotes excitatory activity).

The molecular mechanisms promoted by Li-TMS are similar to those induced by HI-TMS: in both techniques, a repetitive magnetic field is dispensed onto the head of the patient by using a stimulation coil; these repetitive magnetic fields penetrate the skull and reach a specific cortical area. In the case of Li-TMS, no action potential is induced [34]. However, unlike the HI-TMS, when the Li-TMS pulses reach the cerebral cortex, this produces a partial depolarization of the cell membrane and induces an ionic flux to the inside of the neuron (mostly Ca^2+^ ion through L-type voltage-dependent channels), acting as a facilitator of cellular, molecular and genetics changes, observed in in vitro and in vivo investigations [33], in animal and human trials. In addition, it has been shown that Li-TMS increases a) the brain-derived neurotrophic factor (BDNF) in cortical areas, b) nitric oxide (NO, which increases blood flow and promotes angiogenesis processes), and c) neurotransmitters (mostly serotonin and dopamine) and also promotes processes of neurogenesis and neuroplasticity [35-37]. In addition, Li-TMS and PEMF positively affect key neurotransmitters in depression, including serotonin, dopamine, noradrenaline, and acetylcholine, as well as modify the expression of opioid receptors [33]. These changes in neurochemical dynamics can contribute to the relief of depressive symptoms.

Currently, Li-TMS is used to explore its therapeutic effects in different psychiatric pathologies including TRD [38-40], anxiety [41-42], ASD [43], tinnitus [44], insomnia [45], and cerebrovascular events [46]. In the last decade, Li-TMS has gained particular interest due to the cellular effects this promotes in neurons. Li-TMS seems to improve and maintain cellular survival, fostering a new type of growth and BDNF that induces regeneration, and neuroplasticity and regulates molecular activity, despite the subthreshold stimuli [33].

One of the advantages of using Li-TMS is that coils can be designed in different arrangements (including the classical circular and butterfly or eight-shaped coils, and the newest multi-coil designs), which can be applied in both CNS and corporal disorders. Generally, the therapeutic response with Li-TMS takes longer than HI-TMS. However, manufacturing and clinical application costs are significantly lower [47].

This technical report aims to outline the outcomes obtained when using a Li-TMS device in patients with MDD.

## Technical report

Methodology

This retrospective observational technical report is based on data from the comprehensive psychiatric clinic Rome Psiquiatría in Mexico City, which is approved by Mexican health authorities for treating psychiatric disorders (Rome 213300536X0662). The psychiatrists involved hold approval and certification from the Mexican Council of Psychiatry. The study analyzed data collected from 2022 to 2023.

In this technical report, the following inclusion criteria were considered: individuals aged 18 to 65 years willing to participate, with a confirmed diagnosis of treatment-resistant major depressive disorder (which was determined during the initial psychiatric evaluation using DSM-5 criteria [48]), and the subject signature of the consent form. The TRD diagnosis required an inadequate response to two different pharmacological treatments administered at appropriate doses and periods. Medical history and concurrent treatments were also documented. Exclusion criteria were discussed with each participant (subjects with diagnoses other than TRD, subjects with diagnosis and/or medical history of epilepsy or convulsive crisis in the previous year, and subjects with metallic implants in the head).

A total of 41 participants (20 men and 21 women) met the inclusion and exclusion criteria. Concomitant medications for treatment-resistant major depressive disorder remained consistent, with dose adjustments occurring one month before or after Li-TMS treatment sessions. Li-TMS treatment was not accompanied by medication modifications to directly assess its effects. Objective evaluation of depressive symptoms using the Beck Depression Inventory (BDI) [49] occurred before the first session (T1) and at the end of the 10th (T2) and 20th Li-TMS sessions (T3). Adverse events were recorded after each Li-TMS session.

Li-TMS Sessions

The study participants received 20 sessions of Li-TMS with the medical device Nibbot Pro. Each Li-TMS session consisted of positioning an eight-shaped coil over the left dorsolateral prefrontal cortex (DLPFC), which dispensed magnetic pulses with the continuous theta burst pattern at 30 mT for 60 minutes. The 20 Li-TMS sessions were scheduled for each patient daily, on consecutive days between 10:00 a.m. and 5:00 p.m. from Monday to Saturday.

Statistical Analysis

IBM SPSS Statistics for Windows (IBM Corp., Armonk, NY) was utilized for the analysis. Initially, the Kolmogorov-Smirnov and Shapiro-Wilk tests for normality were conducted. Both tests yielded a p-value greater than 0.05 for T1 and T2, indicating that the data for these time points can be considered normally distributed. For T3, however, the p-value was less than 0.05, suggesting a deviation from the normal distribution.

Subsequently, the non-parametric Friedman test was employed, revealing that the difference in average scores among the three-time points was statistically significant, with a p-value less than 0.0001.

The decision to utilize the Friedman test instead of a parametric analysis was justified by the fact that not all groups exhibited normal distribution (specifically, T3). In addition, this choice highlights the importance of considering various aspects of the data structure, including the presence of outliers, homogeneity of variances, sample size, and the consistency of analysis across different groups or measurement times.

Results

**Figure 1:**
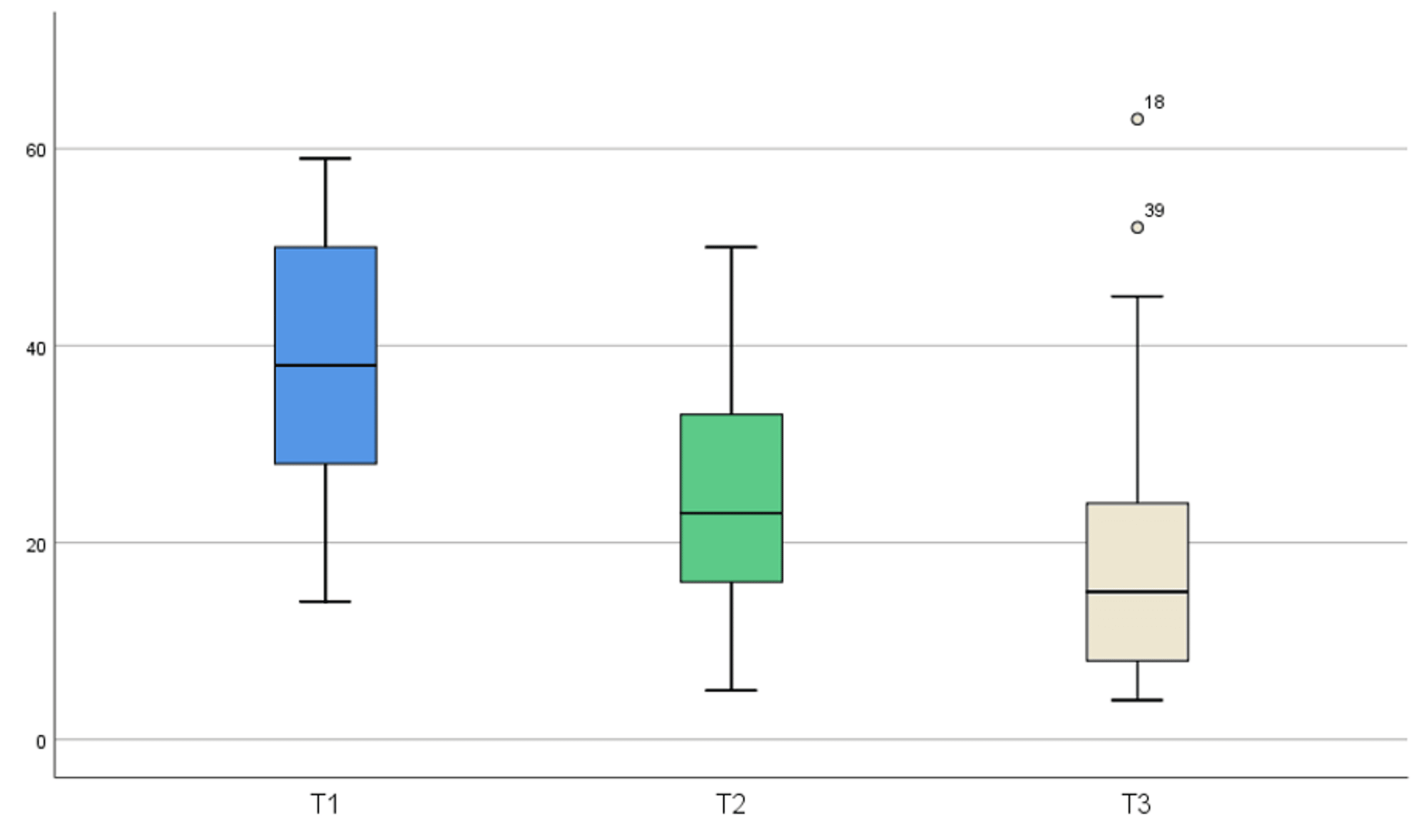
Correlation and distribution of BDI scores at different times of assessment throughout the Li-TMS treatment in patients with TRD. The box plot depicts how the medians and interquartile ranges (IQRs) of the BDI scores decrease in each successive assessment. T1 (BDI assessment before session 1) reflects the highest IQR values in comparison with T2 (BDI assessment at the end of session 10) and T3 (BDI assessment at the end of session 20), which show lower IQR values, with T3 the lowest, suggesting that the Li-TMS treatment applied to TRD patients tends to decrease depressive symptoms over time.

The BDI scores obtained from the clinical evaluations of the participants at sessions 1, 10, and 20 (T1, T2, and T3) are depicted in Figure 1. These results indicate a significant improvement in depressive symptoms throughout the LI-TMS treatment, as evidenced by a decrease in BDI scores from the baseline in comparison with the successive assessments at the end of sessions 10 and 20. In addition, the Friedman test was conducted to determine if there was statistical significance among the three assessment points. The Friedman test, being the non-parametric equivalent of the ANOVA test for repeated measures, is an appropriate choice for comparing three paired groups since it does not require the assumption of normality in the data distribution. The results of the Friedman test applied to the data collected during this study yielded a p-value of 2.32×10^−15^. This p-value is significantly lower than the conventional threshold of 0.05 used to determine statistical significance. This outcome suggests that there is a statistically significant difference among the BDI scores at the different times of assessment, indicating that the variations observed before the initial Li-TMS session (T1), at the 10th Li-TMS session (T2), and in the last session (T3) are not merely due to chance. Instead, this result reflects real changes in BDI scores throughout the Li-TMS sessions.

## Discussion

In this study, the efficacy of the Li-TMS treatment in subjects diagnosed with MDD resistant to treatment (TRD) was evaluated. The sample consisted of 41 participants, evenly divided by gender to represent both sexes equally. The findings of this study indicate promising results for Li-TMS as a therapeutic tool in treating TRD. 

The data collected in this study revealed a significant positive correlation between the BDI scores obtained at different times during the treatment (before the first session and at the end of the 10th and 20th Li-TMS sessions). This outcome suggests that patients who scored either high or low the BDI tended to maintain similar trends throughout Li-TMS treatment. However, it is crucial to highlight the general decrease in medians and interquartile ranges observed in the successive scores. This indicates a relevant clinical improvement in depressive symptoms among the patients, as reported in similar studies where depression scales served as indicators of antidepressant effects [38-40]. Regarding our study, most subjects receiving Li-TMS reported no side effects, with only a few cases experiencing mild and transient headaches during the initial sessions.

The scores obtained before the start of the Li-TMS treatment (T1) showed the highest values, while those at T3 (scores collected at the end of the Li-TMS treatment, after session 20) exhibited a significant reduction. This decreasing trend in BDI scores is a promising indicator of the therapeutic effect of the Li-TMS treatment on depressive symptoms in subjects with TRD. These results show similar trends to other investigations where progressive antidepressant effects were observed in patients with TRD after five weeks of active treatment with transcranial-pulsed electromagnetic fields (T-PEMF) therapy, characterized by a statistical reduction in the Hamilton Depression Scale 17-items (HAMD-17) - including the six-item subscale, HAMD-6 - and the Melancholia Scale (MES) starting on the second week of treatment and reaching response rates (response defined as a 50% or more reduction of the HAMD-17 baseline scores) of 61% at the fifth week, in comparison to the sham group [38]. In addition, our findings build on previous promising results evaluating the remission rates (defined as a score of 7 or less in the HAMD-17) that suggest benefits from add-on treatment with T-PEMF, as evidenced by improvements in depression scores in the HAMD-17 from 20.6 to 11.6 and 20.4 to 6.8, over eight weeks of treatment, which was correlated to a remission rate of ~70% at the eight week [39-40]. The consistency in the decline of scores suggests a significant improvement in mood and cognitive functions among the subjects, which is critical in TRD treatment.

Previous studies, such as Martiny et al. (2010), Strasso et al. (2014), and Larsen et al. (2020), used a variety of tests, such as Fisher’s exact test, Mann-Whitney test, and two-sample t-test, respectively, for their analysis [38-40]. On the other hand, in this study, the Friedman test was used as it allowed us to analyze the differences between BDI scores at different assessment time points confirming the existence of statistically significant differences. This finding reinforces the hypothesis that Li-TMS has a positive and measurable effect on the improvement of depressive symptoms.

The results of this research align with previous studies highlighting the effectiveness of low-intensity PEMF therapy in depression treatment. For instance, Larsen et al. observed that eight-week T-PEMF therapy aided as a beneficial adjunct in patients with treatment-resistant depression, with 49% of the subjects with a non-chronic depressive episode and 28% with a chronic episode showing a favorable response to treatment at the end of the study [39]. Similarly, Martiny and his team demonstrated that the use of T-PEMF in combination with antidepressants is superior to sham treatment in patients with treatment-resistant depression, with clinically and statistically significant improvements from the first weeks of therapy [38]. Regarding the dosage of T-PEMF, the research by Straasø et al. revealed no significant differences in the effectiveness of the treatment for major depression, whether administered once or twice a day after eight weeks of stimulation, with clinical improvement in depressive symptoms observed in both groups [40]. However, it is important to consider that, like any other therapeutic intervention, the efficacy of Li-TMS may vary depending on individual factors, such as the severity of depression, the patient's medical history, and the biological response to treatment.

The limitations of Li-TMS include the need for multiple sessions and the limited availability of equipment in some regions. However, among its strengths lies its efficacy and the lack of systemic side effects compared to medications. The implications suggest a potential shift in the standard treatment for disorders, such as MDD [50]. Future research requires further exploration of the neurophysiological mechanisms involved in therapeutic response. Li-TMS may be a cost-effective option in countries where antidepressant medications are expensive due to their potential to reduce the need for long-term medication and the possibility of decreasing costs associated with the side effects of pharmacological treatments.

## Conclusions

This study offers scientific evidence regarding the safety and efficacy of Li-TMS treatment for depression, laying the groundwork for further research in this area to delve deeper into the underlying mechanisms of this therapy. The consistency in the improvement of the depressive symptoms, coupled with the statistically significant trend in the results and the minimal reported side effects, suggests that Li-TMS should be regarded as a safe valuable therapeutic alternative for depression treatment, especially in countries where access to other antidepressant options may be challenging or costly for patients. However, there are currently no studies that can precisely determine if Li-TMS may be a more affordable option than drug medication. It is certainly true that TMS may represent a higher cost in the short term; however, in the long term, it may lead to significant savings. This is because the patient may discontinue pharmacotherapy and, in the best-case scenario, transition to maintenance TMS sessions. This aspect is still under investigation to evaluate the potential for remission and the frequency of maintenance therapy.
