# Correction: Technical Report: Efficacy and Safety of Low-Intensity Transcranial Magnetic Stimulation in the Remission of Depressive Symptoms in Patients With Treatment-Resistant Depression in Mexico

**DOI:** 10.7759/cureus.c240

**Published:** 2025-08-13

**Authors:** Cristina Rodríguez Hernández, Omar Medrano Espinosa, Raúl Sampieri-Cabrera, Alan R Oviedo Lara

**Affiliations:** 1 Psychiatry, Rome Psiquiatría Integral, Mexico City, MEX; 2 Department of Physiology, Faculty of Medicine, Universidad Nacional Autónoma de México, Mexico City, MEX; 3 Research, Nibbot International, Mexico City, MEX

The Conflicts of Interest statement of this article has been corrected to disclose the following conflicts of interest:

- Payment/services info: Nibbot International provided technical support and critical review of the manuscript prior to submission.
- Financial relationships: Alan R. Oviedo Lara declares employment from Nibbot International.

